# Supplementary material for: Genome-Wide Identification of the NPR1-like Gene Family in Solanum tuberosum and Functional Characterization of StNPR1 in Resistance to Ralstonia solanacearum
Source: Genes (Basel). 2023 May 27;14(6):1170. doi: 10.3390/genes14061170 (PMC10298024; doi:10.3390/genes14061170)
Supplement: Supplementary file 1 [file genes-14-01170-s001.zip › genes-2387179-supplementary.pdf]

Supplementary Table S1

Table S1. Primers used in this research

Name	Accession No.	Sequence (5'→3')	Purpose
StNPR1-FW	XM_006357647.2	CGGTTCTTTCCACGTTGTT	qRT-PCR
StNPR1-RV	XM_006357647.2	GCTTCTTCAGTTGACGCTCT	
StNPR2-FW	XM_006366563.2	GCCCATGAAACTGCTCTAC	qRT-PCR
StNPR2-RV	XM_006366563.2	CAACCTCCCTCAAGTTTCC	
StNPR3-FW	XM_006353007.2	ACTACGCCTCAAATCCAAC	qRT-PCR
StNPR3-RV	XM_006353007.2	ACCCTTCTCCCATTACCAT	
StNPR4-FW	XM_006364369.2	TGTGAACGAGGTACTTGAGC	qRT-PCR
StNPR4-RV	XM_006364369.2	GTATGTCCGTCGCGTGTAG	
StNPR5-FW	XM_006351200.2	CTGAAATCTTAGCGAAACACC	qRT-PCR
StNPR5-RV	XM_006351200.2	GTCAAGGGCTCGTCTCATT	
StNPR6-FW	XM_004249185.4	ATAAGCATGGTGGAGAAAGC	qRT-PCR
StNPR6-RV	XM_004249185.4	AGGGAGGTGTTTGGTTAGGAT	
StNPR1-FW-2	XM_006357647.2	CTTGTCTTGGAGTTTTCAC	Gene clone
StNPR1-RV-2	XM_006357647.2	CCTAAAAGGGAGATTATTGGGC	
StAOS-FW	XM_006366379.2	AGACTTCGTCGTACTGGTTT	qRT-PCR
StAOS-RV	XM_006366379.2	AAGGTAATTGCAGCTCCTAA	
StICS1-FW	XM_015312034.1	ACAATATGGCAGCTTTCCT	qRT-PCR
StICS1-RV	XM_015312034.1	GTCCCAAGACGCTTTACCC	
StPAD4-FW	XM_006350417.2	CGAGCTATGAAGCAGGGAT	qRT-PCR
StPAD4-RV	XM_006350417.2	GCTGTTGAGGTTGCGAGTT	
StPAL-FW	NM_001318638.1	GTGATCTTGTCCCTCTTTCC	qRT-PCR
StPAL-RV	NM_001318638.1	AACGCTTCCTCAGCATTAA	
StPDF1.2-FW	XR_001475358.1	ATGGCGAAGTACACTACTTT	qRT-PCR
StPDF1.2-RV	XR_001475358.1	CGAGAAAAGTAGTGTACTTC	
StPR1-FW	XM_006367029.2	ACACTCTGGTGGCCCTTAC	qRT-PCR
StPR1-RV	XM_006367029.2	ACATACTTTACCCGCTTGA	
StVSP1-FW	XM_015306769.1	CCTCAAATCAACTGCCTCA	qRT-PCR
StVSP1-RV	XM_015306769.1	TGTAGTGCCCGACGTAATC	
StWRKY70-FW	XM_006356415.2	TCCAAAGGACAAAGTGATA	qRT-PCR
StWRKY70-RV	XM_006356415.2	TGACCCCTTCATAATAAGC	
β-actin-FW	X55747	GCTTCCCGATGGTCAAGTCA	qRT-PCR
β-actin-RV	X55747	GGATTCCAGCTGCTTCCATTC	

Supplementary Table S2

Table S2. Characteristics of potato NPR1-like gene family members

	Gene ID	Chr.	Gene position		Gene length (bp)	Protein length (aa)	Mol. Wt. (kD)	pI
			Start	End				
StNPR1	Soltu.DM.07G011890	A07	38273905	38290258	1884	627	69593.25	6.46
StNPR2	Soltu.DM.07G014680	A07	44404274	44408833	1728	575	64579.71	5.66
StNPR3	Soltu.DM.02G012330	A02	27057682	27063283	1746	587	64360.48	6.03
StNPR4	Soltu.DM.04G012100	A04	13482468	13485467	1386	461	50646.89	6.33
StNPR5	Soltu.DM.10G027630	A10	58822974	58825292	1464	487	53415.76	6.14
StNPR6	Soltu.DM.10G027950	A10	59078099	59080668	1473	490	54288.74	6.28

Supplementary Table S3

Table S3. Associated NPR1-like genes in various plants Information

Species	Gene name	Accession number
Oryza sativa	*OsNPR1*	DQ450948
	OsNPR2	DQ450949
	OsNPR3	DQ450952
	OsNPR5	DQ450956
Hordeum vulgare	*HvNPR1*	AM050559
Brachypodium distachyon	*BdNPR1*	XP_003564857
Zea mays	*ZmNPR1*	NP_001354806
Musa acuminata	*MNPR1A*	DQ925843
	MNPR1B	EF137717
Triticum dicoccoides	*wNPR1*	JX424315
Arabidopsis thaliana	*AtNPR1*	At1g64280
	AtNPR2	At4g26120
	AtNPR3	At5g45110
	AtNPR4	At4g19660
	AtNPR5	At2g41370
	AtNPR6	At3g57130
Brassica juncea	*BjNPR1*	ABC94642
Morus multicaulis	*MuNPR1*	JX204735
Nicotiana tabacum	*NtNPR1*	EF988657
Glycine max	*GmNPR1*	FJ418595
Malus domestica	*MpNPR1*	ACC77697.1
Vitis vinifera	*VvNPR1*	XP_002281475
Solanum lycopersicum	*SlNPR1*	APY24056.1
Ipomoea batatas	*IbNPR1*	EF190039
Theobroma cacao	*TcNPR1*	HM117159
	TcNPR3	JX983187
Persea americana	*PaNPR1*	KR056089
	PaNPR2	KR056090
	PaNPR3	KR056091
	PaNPR4	KR056092
	PaNPR5	KR056093
Populus deltoids	*PtNPR1*	XP_024460251
	PtNPR2	XP_024451241
	PtNPR3	XP_002322351
